# Positive Association between Individualism and Vaccination Resistance against COVID-19 Vaccination among Chinese Adults: Mediations via Perceived Personal and Societal Benefits

**DOI:** 10.3390/vaccines9111225

**Published:** 2021-10-21

**Authors:** Yanqiu Yu, Mason M. C. Lau, Joseph Tak-Fai Lau

**Affiliations:** Center for Health Behaviours Research, Jockey Club School of Public Health and Primary Care, The Chinese University of Hong Kong, Hong Kong, China; yuyanqiu@link.cuhk.edu.hk (Y.Y.); mason-lau@cuhk.edu.hk (M.M.C.L.)

**Keywords:** vaccination resistance, COVID-19 vaccination, personal benefits, societal benefits, individualism, China

## Abstract

Background: Vaccination resistance is the key hurdle against herd immunity as it limits the final vaccination coverage. This study investigated the prevalence and factors of COVID-19 vaccination resistance (i.e., those indicating definitely not taking up COVID-19 vaccination), including individualism, perceived personal benefits (PPB) and perceived societal benefits (PSB) of COVID-19 vaccination, and related mechanisms of the association. Methods: A random telephone survey interviewed 395 unvaccinated adults aged 18–75 not having scheduled for COVID-19 vaccination in May 2021 in Hong Kong, China (response rate = 56.8%). Results: The prevalence of vaccination resistance was 56.5%. Adjusted for background factors, individualism, PPB, and PSB were significantly associated with vaccination resistance. Path analysis showed that individualism exhibited a direct effect on vaccination resistance and a 3-step indirect effect (individualism → PSB → PPB→ vaccination resistance) that explained 46.8% of the total effect. The two 2-step indirect paths via PPB only and via PSB only were non-significant. Conclusion: High prevalence of vaccination resistance was observed. Individualism increased vaccination resistance via its direct and indirect effects. Health promotion may emphasize collective good to reduce the impact of individualism and promote PPB/PSB, which may reduce vaccination resistance directly and alleviate the impact of individualism on vaccination resistance indirectly.

## 1. Introduction

The Coronavirus Disease 2019 (COVID-19) pandemic has lasted for over a year, and there is no sign that it will subside in the recent future. Despite the expedited availability of COVID-19 vaccines, vaccine hesitancy is common, with the prevalence ranging from 11.4% to 83.0% in the general population worldwide prior to the rollout of COVID-19 vaccines in December 2020 [1,2,3,4,5,6]. As of October 14, 2021, there were globally more than 6.6 billion doses of COVID-19 vaccine inoculations, while 43% of the global population has received two doses [7]. The coverage varied greatly across countries, ranging from <1% (e.g., Sudan) to >70% (e.g., Canada) [7]. There are many pre-rollout studies regarding inclination toward COVID-19 vaccination. However, the performance of the COVID-19 vaccines (e.g., safety and efficacy) was largely uncertain during that time period, while only a few of them asked about vaccination intention based on hypothetical characteristics of the vaccines [8,9]. Recently, some studies looked at actual COVID-19 vaccination behavior [10,11], but it is still warranted to understand people’s inclinations. During the post-rollout period, the decision on COVID-19 vaccination can refer to the growingly available clinical trial data and post-marketing surveillance reports. It is argued that post-rollout intention data are more predictive of future vaccination rates than pre-rollout data. This study is one of the few studies that was conducted during the early rollout phase (about two months since the rollout) that investigated the subject.

Most of the studies investigating inclination toward vaccination involved terms, such as ‘acceptability’ [12,13], ‘willingness’ [14,15], ‘intention’ [8,16], and ‘hesitancy’ [3,17]. Such concepts and related items used, however, overlapped with each other, and distinctions were blurred. A researcher pointed out that “Vaccine hesitancy is a continuum that rests between acceptance of all recommended vaccines and refusal of all recommended vaccines. However, lack of hesitancy should not be confused with certainty. Some people will still accept vaccines even when they are uncertain or refuse vaccines when they are uncertain” [12]. Better conceptualization of inclination toward vaccination is warranted. Unvaccinated people who have not scheduled the first dose can broadly be classified into three groups in terms of the inclination toward taking up the first dose: (1) those who are willing to or have planned to take up the first dose while having or not having decided when to do so, (2) those who are hesitating and hence have undecided whether to take up the first dose, (3) those who have decided not to take up COVID-19 vaccination. Most of the studies titled ‘willingness’, ‘acceptability’, and ‘intention’ of COVID-19 vaccination were based on binary outcomes, classifying the above-mentioned Group 1 versus Groups 2 and 3 as inclining versus not inclining toward vaccination [18]. Groups 2 (hesitancy) and 3 (resistance) are, however, different in nature and require different health promotion strategies. A small number of pre-rollout studies specifically looked at the third group, i.e., vaccination resistance or refusal (e.g., ‘absolutely against taking up vaccination’) [19,20]. The present study is one of the few studies investigating vaccination resistance during the post-rollout period, i.e., comparing people indicating that they would definitely not take up COVID-19 vaccination (the resistant group) versus other people (the non-resistant group). The size of the resistant group (Group 3) is certainly a major determinant of the final COVID-19 vaccination coverage. The size of the hesitant group (Group 2) is another contributor. However, it is expected that a much larger proportion of the hesitant group than the resistant group would eventually take up COVID-19 vaccination as many of the latter were holding a wait-and-see attitude and might take up vaccination when they know more about the vaccines’ efficacy and safety [8]. A remark is that ‘compulsory’ vaccination, which has been applied to specific populations in multiple countries in explicit and subtle forms, would diminish the impact of vaccination resistance on the final vaccination coverage, while lack of vaccine supply would diminish the impact of Group 1 and Group 2 on the final coverage. So far, no countries have applied global compulsory measures to the entire general population.

Health economists suggest that health-related decisions are heavily driven by perceived gains (rewards) and losses [21]. Perceived benefits is a key construct of the Health Belief Model [22] and was positively associated with many health-protective behaviors, including COVID-19 vaccination intention and influenza vaccination [23,24]. Notably, perceived benefits of COVID-19 vaccination include perceived personal benefits (PPB) and perceived societal benefits (PSB). Examples of the former include self-protection and relief from worries about infection and restrictions in accessing some public venues because of the unvaccinated status, while examples of the latter include the establishment of herd immunity and speeding up economic recovery. It was expected in this study that PSB would be significantly associated with PPB. Such perceptions were expected to be positively associated with vaccination intention/behavior [9,25] and hence negatively associated with vaccination resistance.

While PPB and PSB are individual-level factors, socio-cultural factors are important parts of the structural determinants of health-related behaviors, according to the socio-ecological model [26]. Collectivism is particularly relevant in understanding COVID-19 vaccination, as COVID-19 and COVID-19 vaccination have simultaneous and multi-dimensional impacts on individuals, their surrounding people, and society at large. Collectivism refers to regarding people as inter-dependent individuals of the society so that individual decisions are often driven by others’ interests [27]. COVID-19 vaccination, like influenza vaccination, may be affected by collectivism, as it can be regarded as a prosocial behavior [28]. From another perspective, collectivism may increase COVID-19 vaccination as it is a form of social capital [29], which, in general, predicts beneficial collective community efforts, including COVID-19 preventive behaviors [30] and COVID-19 vaccination intention [31].

Individualism and collectivism are at the opposite ends of the spectrum. Individualism refers to seeing individuals as being largely independent of each other so that people’s decisions are heavily driven by self-interest [27]. According to Triandis’ cultural framework, individualism is an important part of the cultural environment that shapes an individual’s beliefs, attitudes, norms, values, and ways of thinking [27]. It may hence affect perceptions related to health behaviors. There are reasons to believe that individualism would be associated with COVID-19 vaccination, as individualism was negatively associated with the uptake of preventive measures amid the COVID-19 pandemic [32,33]. For instance, a study of five cross-sectional surveys conducted in the U.K. reported that an individualistic worldview was associated with fewer COVID-19-related protective behaviors (e.g., social distancing and facemask wearing) [32]. Furthermore, individualists tend to emphasize more on personal freedom, while perceived infringement of personal freedom related to COVID-19 preventive measures was negatively associated with the adoption of such measures [34]. Severe confrontations and protests against strict public COVID-19 measures (e.g., lockdown and facemask use) have occurred in multiple countries [35]. More and more countries are attempting to exercise mandatory COVID-19 vaccination in special groups (e.g., healthcare workers) [36]. Individualists might object to such policies and possess anti-vaccination perceptions. The role of individualism in affecting COVID-19 vaccination (and any form of vaccination) has not been investigated. The hypothesis that individualism would be positively associated with vaccination resistance was tested in the present study.

It is interesting to understand the mechanisms underlying the potential association between individualism and vaccination resistance. Numerous researchers have suggested that dispositional traits are distal factors of health behaviors/outcomes, while health beliefs are proximal factors that mediate the associations between distal factors and health behaviors [37,38]. As individualism is a trait [27] and perceived benefits (PPB/PSB) of COVID-19 vaccination are health beliefs, PPB/PSB may suppress/mediate between individualism and vaccination resistance. As individualists were more likely than collectivists to pay more attention to gains resulting from an action (perceived benefits of COVID-19 vaccination in this case) [39], individualism might increase PPB. In parallel, individualists might perceive fewer societal benefits of COVID-19 vaccination than collectivists, as they may be less concerned about benefits that are not self-directed. As aforementioned, literature has consistently found significant associations between perceived benefits of COVID-19 vaccination and vaccination intention [9,23]. Under such circumstances, it is expected that PPB and PSB would fully or partially explain the mechanisms between individualism and vaccination resistance via suppression by PPB and/or via mediation by PSB, respectively. Furthermore, as PSB was expected to be associated with PPB, its effect on vaccination resistance may include an indirect effect via PPB, in addition to a direct effect from PSB to vaccination resistance.

Given the background, the present study investigated the level of vaccination resistance among unvaccinated adults who had not scheduled for the first dose of COVID-19 vaccines in the general adult population in Hong Kong, China. The associations between individualism/perceived benefits of COVID-19 vaccination (PPB and PSB) and vaccination resistance were tested. Furthermore, the mechanisms between individualism and vaccination resistance were explored. It was hypothesized that in addition to the direct positive effect from individualism to vaccination resistance, there are two possible 2-step indirect paths and one 3-step indirect path: (a) PPB would suppress between individualism and vaccination resistance (i.e., higher individualism → higher PPB → lower vaccination resistance). (b) PSB would mediate between individualism and vaccination resistance (i.e., higher individualism → lower PSB → higher vaccination resistance). (c) Individualism would reduce PSB; the reduction in PSB would then reduce PPB; the reduction in PPB would then heighten vaccination resistance (i.e., individualism → lower PSB → lower PPB → higher vaccination resistance).

## 2. Materials and Methods

### 2.1. Participants and Data Collection

A random telephone survey was conducted in a Chinese population aged 18–75 years in Hong Kong, China, from 14 to 27 May 2021. As we investigated vaccination resistance among unvaccinated adults who had not scheduled to take up the first dose, those who had taken up one or two doses of COVID-19 vaccination and those who had scheduled to take up the first dose were excluded from data analysis.

Telephone numbers were randomly selected from updated landline telephone directories by using a computer program; about 500,000 residential telephone numbers were thus used as seed numbers. To solicit potentially unlisted numbers, three extra ‘phone’ numbers were generated for each of the listed telephone numbers by randomizing the last two digits of the listed numbers, which were mixed with the original 500,000 phone numbers after removal of duplications. This pool of numbers comprised the sampling frame, from which random numbers were drawn for the interviews. Invalid numbers (non-residential numbers (commercial numbers), fax numbers, and those involving at least three unanswered telephone calls made on different days/hours) were replaced by additional random numbers.

The telephone survey was administered between 5 and 10 pm to avoid over-sampling non-working individuals. Telephone numbers were randomly drawn from the most updated residential telephone directory. Unanswered telephone calls were given at least three attempts. The eligible household member whose birthday was closest to the survey date was interviewed. Appointments were made if necessary. Trained interviewers briefed the participants about the study, sought their verbal informed consent, and signed a form pledging completion of the required consent procedures. No incentives were given to the participants. Participants could quit any time without being questioned. Ethics approval was obtained from the corresponding author’s affiliated institution (Ref No. SBRE-20-722).

As seen from the flow chart of participant recruitment (Supplementary Figure S1), a total of 5023 telephone numbers were sampled, of which 4143 were invalid numbers (e.g., non-residential numbers, empty numbers, and no eligible persons). The remaining phone numbers reached 880 eligible households, defined as those having at least one eligible prospective participant in the household who had been contacted by the interviewer; 500 interviews were completed. The response rate, defined as the number of completed interviews (500) divided by the number of eligible contacts (880), was 56.8%. Of all the 500 participants, 105 (21.0%) were excluded from this study as they had taken up at least one dose of COVID-19 vaccination (n = 81) or made an appointment to take up the first dose (n = 24). The effective sample size was 395.

### 2.2. Measurements

#### 2.2.1. Background Information

Information about socio-demographics (sex, age, educational level, and marital status), chronic disease status, and history of influenza vaccination was collected.

#### 2.2.2. Vaccination Resistance

Among the unvaccinated individuals who had neither taken up vaccination nor made an appointment to take up the first dose of COVID-19 vaccination, participants were asked whether they were definitely not going to take up COVID-19 vaccination in the future (yes/no).

#### 2.2.3. PPB of COVID-19 Vaccination

Eleven items were self-constructed to assess the level of four domains of PPB, including (1) three items regarding perceived physical benefits (your taking up COVID-19 vaccination would protect yourself from COVID-19 infection, protect your family members from COVID-19 infection, and reduce the risk of severe side effects and deaths in case of COVID-19 infection, respectively), (2) four items assessed perceived practical benefits (your taking up COVID-19 vaccination would facilitate visiting public venues, facilitate traveling with ‘vaccine passports’, fulfill work requirement, and restore ‘normal’ life comparable to the pre-COVID-19 period, respectively), (3) two items assessed emotional benefits (your taking up COVID-19 vaccination would relieve worries about COVID-19 infection and relieve worries about severe side effects in case of COVID-19 infection, respectively), and (4) two items regarding interpersonal benefits (your taking up COVID-19 vaccination would remove social pressure toward COVID-19 vaccination and increase the participants’ friends’ willingness to have social gatherings with him/her). The 5-point Likert rating scales were used for all the items (1 = totally disagree to 5 = totally agree). Cronbach’s alpha of the summative scale of PPB adding up all the items was 0.93 in this study. Similar items have been used in a number of previously published studies [9,25,40,41].

#### 2.2.4. PSB of COVID-19 Vaccination

Three items were self-constructed to assess the level of PSB, including those of your taking up COVID-19 would (a) control the local COVID-19 outbreak, (b) facilitate prompt local economic recovery, and (c) facilitate removal of travel restrictions between Hong Kong and mainland China (e.g., quarantine requirements), respectively. The 5-point Likert rating scales were used (1 = totally disagree to 5 = totally agree). Cronbach’s alpha of the summative scale adding up the three items was 0.96 in this study. Similar items have been used in a number of previously published studies [9,42].

#### 2.2.5. Individualism

The level of individualism was assessed by a 4-item individualism subscale of the Individualism and Collectivism Scale (ICS) [43]. The ICS has been validated in countries such as the U.S. and South Korea [43]. Two independent bilingual researchers translated the English version into Chinese, which was back-translated into English, and the final Chinese version was finalized by the corresponding author of this study. The four items were “I’d rather depend on myself than others”, “I rely on myself most of the time; I rarely rely on others”, “I often do ‘my own thing’”, and “My personal identity, independent of others, is very important to me”. The items were rated on 9-point Likert scales (1 = never or definitely no to 9 = always or definitely yes). Higher scores indicated higher levels of individualism. The Cronbach’s alpha was 0.84 in this study.

### 2.3. Data Analysis

The sample size planning was conducted by using the Logistic Regression module in the PASS 11.0. Assuming the prevalence of vaccination resistance ranged from 20% to 70%, the sample size of 395 would have the smallest detectable odds ratio (OR) of 1.41 to 1.48 (power of 0.80 and alpha of 0.05, two-sided) when these individuals were compared with those with the value of the independent variable equal to mean plus one standard deviation. Notably, the sample size required to obtain sufficient power depends on population characteristics (e.g., the strength of the associations) and the acceptable levels of Type I and Type II errors, but not on the population size [44]. The sample size was thus adequate.

Univariable and multivariable (adjusted for background factors) logistic regression analyses were conducted to test the associations between three potential factors (i.e., individualism, PPB, and PSB) and vaccination resistance. Crude odds ratio (ORc), adjusted odds ratio (ORa), and respective 95% confidence intervals (CIs) were generated. Pearson correlation coefficients among the potential factors of vaccination resistance were derived. Path analysis using weighted least square mean and variance adjusted (WLSMV) estimator was conducted to test the suppression/mediation mechanisms of PPB/PSB between individualism and vaccination resistance. Bootstrapping approach (n = 2000) was used to test the significance of the mediation/suppression effect; an indirect effect was considered statistically significant if the 95% CI did not include zero. SPSS 23.0 and Mplus 7.0 were used to conduct data analysis. Statistical significance was defined as *p* < 0.05 (two-tailed).

## 3. Results

### 3.1. Descriptive Statistics

Of all the participants, over half were female (n = 249; 63.0%), aged 18–50 years (n = 225; 57.0%), and currently married (n = 254; 64.3%). About one-fourth had attained an educational level of college or above (n = 109; 27.6%), self-reported having at least one of the listed chronic diseases (n = 108; 27.3%), and had ever taken up influenza vaccination (n = 92; 23.3%) (Table 1). The mean (SD; range) scores of individualism, PPB, and PSB were 24.6 (SD = 5.6; range: 8–36), 3.2 (SD = 0.9; range:1–5), and 3.4 (SD = 1.0; range: 1–5), respectively. The prevalence of vaccination resistance was 56.5% (n = 223; 95% CI: 51.6%, 61.4%) (Table 1).

### 3.2. Factors of Vaccination Resistance

Adjusted for the background factors, the multivariable logistic regression analyses found that individualism was positively associated with vaccination resistance (ORa = 1.05; 95% CI: 1.01, 1.09), while PPB (ORa = 0.40; 95% CI: 0.29, 0.55) and PSB (ORa = 0.61; 95% CI: 0.49, 0.76) were negatively associated with vaccination resistance (Table 2).

### 3.3. Correlation Analyses

Individualism was negatively correlated with PSB (r = −0.19; *p* < 0.001) but not with PPB (r = −0.07; *p* = 0.179). PPB was positively correlated with PSB (r = 0.76; *p* < 0.001).

### 3.4. Path Analysis

**Figure 1:**
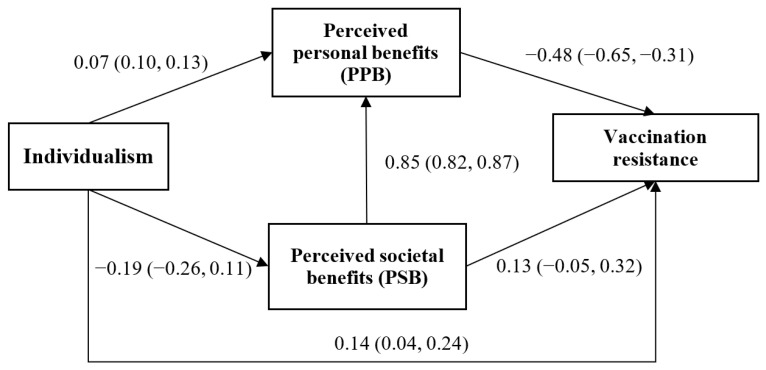
Path analysis of the mechanisms between individualism and vaccination resistance (Standardized beta coefficients and respective 95% confidence intervals were reported).

Figure 1 presents the mechanisms underlying the association between individualism and vaccination resistance via the two types of perceived benefits of COVID-19 vaccination (PPB and PSB). (1) The direct positive effect from individualism to vaccination resistance was statistically significant (β = 0.14; 95% CI: 0.04, 0.24). (2) The two 2-step indirect path between individualism and vaccination resistance via PPB only (β = −0.03; 95% CI: −0.07, 0.00) and via PSB only (β = −0.03; 95% CI: -0.06, 0.01) were both statistically non-significant. (3) The 3-step indirect path (i.e., individualism → PSB → PPB → vaccination resistance) was statistically significant (β = 0.07; 95% CI: 0.03, 0.12) and explained 46.8% of the total effect. Both the significant direct and 3-step indirect paths suggested that stronger individualism would result in stronger vaccination resistance. Furthermore, as seen in Figure 1, two of the six paths were statistically non-significant: the association between individualism and PPB (β = 0.07; 95% CI: 0.01, 0.13; *p* = 0.056) and that between PSB and vaccination resistance (β = 0.13; 95% CI: −0.05, 0.32; *p* = 0.231). Thus, PSB had no direct effect on vaccination resistance; it affected vaccination resistance indirectly via PPB, i.e., PSB increased PPB, which, in turn, reduced vaccination resistance.

## 4. Discussion

In summary, the present study observed a high prevalence of vaccination resistance of 57% among unvaccinated Hong Kong adults not having scheduled for the first dose of COVID-19 vaccination. It found that individualism might increase vaccination resistance via a direct effect plus a 3-step indirect effect involving perceived benefits of vaccination. The 3-step path, which explained about half of the total effect, indicated that individualism would firstly increase PSB; the increase in PSB would lead to a reduction in PPB; the reduction in PPB would then enhance vaccination resistance.

As of 27 May 2021 (the last survey date of this study), about 32.8% of the general population in Hong Kong had received at least one dose of COVID-19 vaccination [45]. The finding hence implies that 38.3% of all the adults in Hong Kong [57% × (100% − 32.8%) = 38.3%] might resist COVID-19 vaccination. These people might change their minds, but it is apparent that a majority of them would not take up COVID-19 vaccination eventually. Although the vaccination rate in Hong Kong has been increasing over time, the observed increase might largely reflect actions taken by those who had planned to take up COVID-19 vaccination and those who used to ‘wait and see’ (68.2% of the adult population in Hong Kong held wait-and-see attitude [8]) but not those with resistance. Thus, the increase in vaccination rate might decelerate when the majority of these two groups (planning and waiting) have taken up vaccination, as most of the remaining unvaccinated people would be ‘resisters’. The attainment of a vaccination coverage rate of 70% to 80% (the expected requirement of herd immunity) hence remains a challenge. A policy implication is that to attain herd immunity, it is very important to reduce vaccination resistance, as it determines the final coverage rate.

Our literature review found four studies conducted in the U.K., the U.S., Australia, and Japan, which investigated vaccination resistance (or refusal) in the general adult population [12,14,18,19,20]. The prevalence only ranged from 5.5% to 16.3% and was much lower than that reported in the present study. Except for Japan, these countries have achieved a high prevalence of vaccination [7]. However, since these studies did not exclude vaccinated individuals as they were conducted during pre-rollout periods and different measurements were used, direct comparisons are not feasible. Research about vaccination resistance during post-rollout periods is warranted.

One of the important novel findings is that individualism was positively associated with vaccination resistance. It agrees with Triandis’ cultural framework [27] and corroborates the previously reported negative associations between individualism and COVID-19 preventive behaviors (e.g., social distancing) [32,33]. An experimental study compared two promotion strategies [46]. The first one used a pure individualistic strategy concerning individuals’ perceived risk or cost prior to making vaccination decisions, while the second one focused on individuals’ contribution to the community before making vaccination decisions; the results showed that the communities could achieve a higher vaccination rate at lower cost by using a combined individual/community-based vaccination strategy rather than a purely individualistic strategy [46]. Thus, although individualism, being a trait, seems hard to change, it is possible to ‘neutralize’ its effect on COVID-19 vaccination resistance by emphasizing collective good and the prosocial nature of COVID-19 vaccination, which have been shown to be positively associated with intention to take up COVID-19 vaccination in the next six months [16].

The present study revealed the underlying mechanisms between individualism and vaccination resistance for the first time. It involved a negative direct effect and a partial 3-step indirect effect via PSB then via PPB, which explained 46.8% of the total effect. Both the direct and indirect effects indicated that individualism might increase vaccination resistance. It is not surprising that PPB and PSB were significantly and negatively associated with vaccination resistance. The findings are supported both theoretically [22] and empirically [10,23]. The novelty of the present study is that it considered the effect of both PPB (self-interest) and PSB (others’ interest), which were strongly correlated with each other, and their roles in explaining the potential effect of individualism on vaccination resistance. The findings suggest that modifications of PPB and PSB together may reduce vaccination resistance, as the 3-step indirect effect was significant and showed a large effect size. As the levels of these two types of perceived benefits were only moderate (3.2 and 3.4 out of five, respectively), there is substantial room for improvement. To increase PPB and PSB, health promotion may provide more information about various domains of potential benefits of COVID-19 vaccination, including protection from infection and severe side effects in the case of COVID-19 infection (physical benefits), psychological relief (emotional benefits), facilitation of daily life and travels (practical benefits), and the achievement of herd immunity (societal benefits).

Some details are noteworthy. Individualism was not significantly associated with PPB in the simple correlation, but the *p*-value of the path (shown in the path analysis) from individualism to PPB was 0.056, which was close to the significance level of 0.05. It might have become statistically significant given the larger sample size. As PPB was strongly associated with PSB, the association between individualism and PPB seems to have been suppressed by PSB, which was negatively associated with individualism. In plain language, the association between individualism and PPB may become significant if the effect of PSB was removed, which was supported by the almost significant association between individualism and PPB according to path analysis. Furthermore, it seems that PSB’s effect on vaccination resistance operated only indirectly via its influence on PPB, as the direct path of PSB → vaccination resistance was non-significant. The significant pathway of PSB → PPB → vaccination resistance is understandable, as most of the personal benefits have to be derived from changes in governmental control policies that have important direct bearings on societal benefits (e.g., loosening travel restrictions).

The findings have important implications for health promotion. The high prevalence of vaccination resistance needs to be reduced. Health promotion of perceived benefits (PSB/PPB) may reduce the impact of individualism on vaccination resistance. Furthermore, as informed by the trans-theoretical model (TTM), different strategies are required to initiate behavioral changes among people of different stages of change [47]. Analogically, those planning to take up COVID-19 vaccination were in the preparation stage; hesitant people are positioned between the pre-contemplation and the contemplation stage; ‘resisters’ may belong to the pre-contemplation stage. To move people to higher stages of change, those in the preparation stage need enhancement in self-efficacy (e.g., convenience) and goal setting (e.g., appointment) and to shift the decision balance by emphasizing removal of barriers (e.g., side effects) [47]. Those in the pre-contemplation stage, however, need convincing information about the pros (e.g., PPB and PSB) and cons (e.g., side-effect) of the behavior while emphasizing more on the pros (e.g., protection) to shift the decision balance [47]. Segmentation is an effective strategy of social marketing [48]. The TTM echoes that clear information (evidence), especially that on the benefits of COVID-19 vaccination, would be essential for reducing the size of the ‘resistant segment’. It is also important to pay attention to the quantity, quality, and content of the messages as such information environment may affect one’s willingness to accept COVID-19 vaccination [49].

The present study has several limitations. First, due to the cross-sectional design, temporal and causal relationships cannot be inferred. Second, social desirability bias may exist as COVID-19 vaccination is a socially desirable behavior, and participants might tend not to report vaccination resistance. Third, the characteristics of those participants and refusers may differ, although the response rate of this study (56.8%) was comparable to other local telephone surveys. Fourth, although the age distribution of this study sample was comparable to that of the 2019 Hong Kong census data, the proportion of females may be slightly higher than that of the census. Furthermore, this sample included people aged 18–75 who were classified into the younger (18–50) and older (51–75) age groups, as such age groups showed substantial differences in vaccination intention [8,23]. We did not include the >75 age group due to the belief that the older population may hold different perceptions about the severity of COVID-19, risk of infection, and severity of side effects. In Hong Kong, this group has a much lower vaccination rate than the other age groups [50]. The determinants might be different and require a separate investigation. Notably, it is a limitation that we did not ask about vaccine hesitancy explicitly. Instead, we asked whether the participants would definitely not take up COVID-19 vaccination. Thus, although we have conceptually outlined the three groups, our study did not separate Groups 1 and 2 explicitly. In addition, the present study did not investigate collectivism, which is the contrast of individualism; future studies are warranted to compare the directions and relative effects of individualism and collectivism on COVID-19 vaccination resistance.

## 5. Conclusions

In conclusion, the prevalence of vaccination resistance was alarmingly high among unvaccinated Hong Kong Chinese adults not scheduled to take up the first dose, which was significantly associated with individualism and perceived benefits of COVID-19 vaccination (i.e., PPB and PSB). Modifications of such variables may reduce vaccination resistance and facilitate the achievement of herd immunity. Furthermore, this study revealed the mechanisms between individualism and vaccination resistance via a three-step path (i.e., individualism → PSB → PPB → vaccination resistance). It suggests that individualism may shape the perceived benefits of COVID-19 vaccination, which may, in turn, affect vaccination decisions. The significant direct effect of individualism on vaccination resistance implies that other potential mediators/suppressors may exist but were not investigated in this study (e.g., perceived risk). Thus, future longitudinal studies are warranted to confirm these findings across countries and explore additional potential mediators/moderators between other socio-cultural orientations and various types of COVID-19 vaccination outcomes.

## Figures and Tables

**Table 1:** Participants’ characteristics (n = 395).

	n	%
**Background Factors**		
Sex		
Female	249	63.0
Male	146	37.0
Age groups (years)		
18–50	225	57.0
51–75	170	43.0
Educational attainment		
Below college	278	70.4
College or above	109	27.6
Missing data	8	2.0
Marital status		
Others (single/separated/divorced/w-idowed/cohabited)	141	35.7
Married	254	64.3
Chronic disease status ¶		
No	287	72.7
Yes	108	27.3
History of influenza vaccination		
No	303	76.7
Yes	92	23.3
**Vaccination resistance**		
No	172	43.5
Yes	223	56.5

¶, Chronic disease status was defined as those who self-reported having at least one of the listed common chronic diseases, including hypertension, diabetes, chronic pulmonary diseases, myocardial infarction, cardiac failure, cerebrovascular diseases, Alzheimer’s disease, ulcerative diseases, liver diseases, and tumors.

**Table 2:** Factors of vaccination resistance (n = 395)

	Vaccination Resistance	
	ORc (95% CI)	ORa (95% CI)
Individualism	1.04 (1.00, 1.08) *	1.05 (1.01, 1.09) *
Perceived personal benefits (PPB)	0.51 (0.40, 0.66) ***	0.40 (0.29, 0.55) ***
Perceived societal benefits (PSB)	0.61 (0.49, 0.75) ***	0.61 (0.49, 0.76) ***

ORc = Crude odds ratio; ORa = Adjusted odds ratio; CI = Confidence interval. *, *p* < 0.05; ***, *p* < 0.001. The adjusted models were adjusted for sex, age groups, educational level, marital status, chronic disease status, and history of influenza vaccination.

## Data Availability

The data was available on reasonable request.

## Supplementary Materials

The following are available online at www.mdpi.com/article/10.3390/vaccines9111225/s1, Figure S1: Flowchart of participant recruitment.
