# Supplementary figures and images for: Positive Association between Individualism and Vaccination Resistance against COVID-19 Vaccination among Chinese Adults: Mediations via Perceived Personal and Societal Benefits
Source: Vaccines (Basel). 2021 Oct 21;9(11):1225. doi: 10.3390/vaccines9111225 (PMC8622482; doi:10.3390/vaccines9111225)

## Supplementary Materials

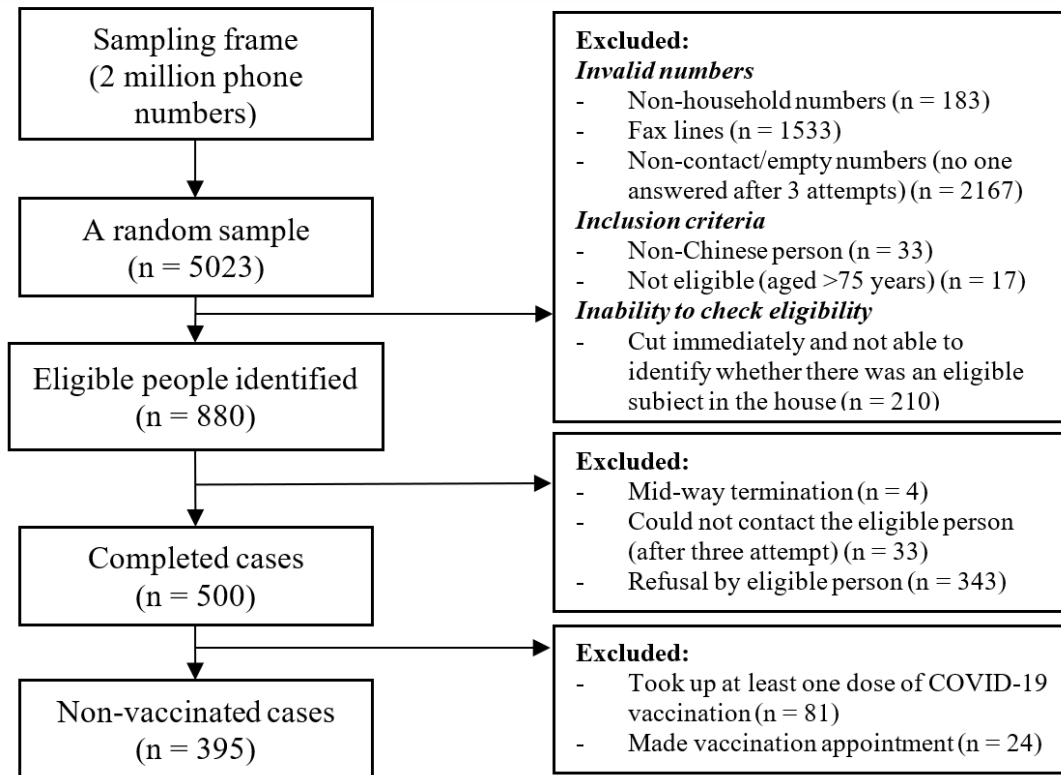

Figure S1. Flowchart of participant recruitment.

Supplement: Supplementary file 1 [file vaccines-09-01225-s001.zip › vaccines-1398964-supplementary.pdf]
